# Prevalence and burden of headache disorders in Lithuania and their public-health and policy implications: a population-based study within the Eurolight Project

**DOI:** 10.1186/s10194-017-0759-5

**Published:** 2017-05-04

**Authors:** Daiva Rastenytė, Dalia Mickevičienė, Lars Jacob Stovner, Hallie Thomas, Colette Andrée, Timothy J Steiner

**Affiliations:** 1 Lithuanian University of Health Sciences, Kaunas, Lithuania; 2 Department of Neuromedicine and Movement Science, Faculty of Medicine and Health Sciences, NTNU Norwegian University of Science and Technology, Trondheim, NO-7941 Norway; 3 Norwegian Advisory Unit on Headache, St Olavs University Hospital, Trondheim, Norway; 4 Department of Pharmaceutical Sciences, University of Basel, Basel, Switzerland; 5 Division of Brain Sciences, Imperial College London, London, UK

**Keywords:** Migraine, Tension-type headache, Medication-overuse headache, Burden, Disability, Lost productivity, Population-based study, Public health, Health policy, Global campaign against headache, Lithuania

## Abstract

**Background:**

The Eurolight project assessed the impact of headache disorders in ten EU countries, using the same structured questionnaire but varying sampling methods. In Lithuania, sample selection employed methods in line with consensus recommendations for population-based burden-of-headache studies.

**Methods:**

The survey was cross-sectional. We identified, from the Residents’ Register Service, a sample of inhabitants of Kaunas city and surrounding Kaunas region reflecting age (in the range 18–65 years), gender and rural/urban distributions of Lithuania. Medical students called unannounced at their homes and conducted face-to-face interviews employing a structured questionnaire.

**Results:**

Of 1137 people in the pre-identified sample, 573 (male 237 [41.4%], female 336 [58.6%]; mean age 40.9 ± 13.8 years) completed interviews (participation proportion: 50.4%). Gender-adjusted 1-year prevalences were: any headache 74.7%; migraine 18.8%; tension-type headache (TTH) 42.2%; all headache on ≥15 days/month 8.6%; probable medication-overuse headache (pMOH) 3.2%. Migraine (OR: 3.6) and pMOH (OR: 2.9) were associated with female gender. All headache types except TTH were associated with significantly diminished quality of life. Migraine caused a mean 4.5% loss in paid worktime per affected male and 3.5% per affected female. Lost per-person times due to TTH were much less, but to pMOH and other headache on ≥15 days/month much higher. Among the entire workforce, lost productivity to migraine was estimated at 0.7%, to TTH 0.3% and to pMOH or other headache on ≥15 days/month 0.5%. The total of 1.5% may translate directly into lost GDP. Alternative calculations based on headache yesterday (with little recall error) produced, for all headache, a corroborating 1.7%. Similar losses from household work would also drain the nation’s economy. Our findings were comparable to those from earlier studies using similar methods in Russia and Georgia.

**Conclusions:**

The multiple burdens from headache in Lithuania indicate substantial ill-health and unmet need for health care. The heavy burdens on individuals are matched by heavy economic burden. Of particular concern is the high prevalence of headache on ≥15 days/month, seen also in Russia and Georgia. Health policy in Lithuania must heed WHO’s advice that effective treatment of headache, clearly desirable for its health benefits, is also expected to be cost-saving.

## Background

The World Health Organization (WHO) recognised headache disorders as major contributors to public ill health, and therefore a public-health priority, in 2000 [1, 2]. Among these disorders, migraine and tension-type headache (TTH) are often lifelong illnesses which, as well as causing pain and disability, also diminish productivity, hinder family and social relationships and impoverish quality of life (QoL). Medication-overuse headache (MOH), usually a *sequela* of migraine or TTH caused by mistreatment of one or the other, occurs by definition on most days and is especially damaging to QoL.

Epidemiologically, many published studies demonstrate that headache disorders are ubiquitous and common [3]. Most of these studies, however, have focused on prevalence to the exclusion of burden. Consequently, they provide data of only limited value for purposes of health policy.

The Global Campaign against Headache was launched in 2004 [4]. Conducted by *Lifting The Burden* (LTB), a UK-registered non-governmental organisation in official relations with WHO [5], its first priority was to fill the major gaps in knowledge of the global burden of headache [6, 7]. For this purpose, LTB developed standardised methodology for population-based studies [8, 9], supported such studies in countries around the world [10], and contributed the data to various iterations of the Global Burden of Disease (GBD) study [11–13]. Migraine became recognized as the sixth highest cause of disability worldwide [12] (third in men and women aged under 50 years [14, 15]), MOH as 18^th^ and headache disorders collectively as third [12, 14]. TTH, relatively non-disabling, is nonetheless now known to be the second most prevalent disorder in the world [12].

In Europe, meanwhile, despite many studies reporting migraine prevalence, there were still major knowledge gaps regarding TTH and MOH, and for all headache types there were few published data on headache-attributed burden [16, 17]. Furthermore, eastern Europe, and especially the countries of the former USSR, were very poorly represented among these [16, 17]. LTB addressed this with population-based studies in Georgia [18, 19] and Russia [20, 21], and Lithuania was included in the Eurolight Project [22].

Eurolight commenced in May 2007 as a partnership activity within the Global Campaign against Headache. Its purpose was to inform policy in the European Union (EU) by assessing the impact of headache. It looked at the EU as a whole, and applied modified cluster-sampling to it with samples drawn from 10 countries [22]. Multiple scientific and lay organisations collaborated with headache experts in these countries, and not all were able to draw population-based samples. In Lithuania alone, the survey was conducted in a sample drawn from public records to be representative of the national population, with each selected participant visited at home. These procedures came close to matching the recommended methodology for population-based burden-of-headache studies [8]. For this reason, we present the Lithuania findings as national estimates.

## Methods

### Study design

The detailed methods of the Eurolight Project have been published elsewhere [22]. We summarise them here. Eurolight was a cross-sectional survey of randomly-selected adults aged 18–65 years, using a structured questionnaire.

The survey in Lithuania was conducted from 1^st^ December, 2009 to 31^st^ March, 2010.

### Sampling

In Lithuania, the survey was population-based, conducted among inhabitants of Kaunas city, the second largest city of Lithuania, and the surrounding areas of Kaunas region in the central part of the Republic. The population of Kaunas region exceeds 420,000 (about 14% of the national population), of whom >90% are ethnic Lithuanians; 80% live in the city and 20% in its surroundings. From the Residents’ Register Service, at the time under the auspices of the Ministry of the Interior but now the Ministry of Justice [23], we identified a sample (*N* = 1,137) reflecting age (in the range 18–65 years) and gender distributions in Lithuania and the proportions living in rural (33%) or urban (67%) areas.

Seven trained medical students from the Lithuanian University of Health Sciences visited the home of each person in this sample, calling unannounced (“cold-calling”). At each house, only the person who had been pre-identified was eligible to participate. Interview was subject to that person’s availability and willingness to participate; if the eligible person was not found during the first visit, at least one additional attempt was made to conduct the interview before that person was regarded as a non-participant.

### Questionnaire

The development, content and validation of the Eurolight structured questionnaire, a close derivative of the HARDSHIP questionnaire [9], have been described previously [24]. The original English version was translated into Lithuanian following LTB’s translation protocol for lay documents [25].

Demographic questions were followed by neutral screening questions for headache (“Have you ever had a headache?” and “Have you had a headache during the last year?”) and, in those screening positively, by headache-diagnostic questions based on ICHD-II [26]. We also asked about headache on the preceding day (“headache yesterday” [HY]). Finally, there were several question sets addressing various components of burden, including symptom burden. As part of the enquiry into burden, we incorporated the HALT questionnaire [27] and WHOQoL-8 [28] as modules in our questionnaire. We applied WHOQoL-8 to all participants, whether reporting headache or not.

Burden enquiry focused primarily on the preceding 3 months (except WHOQoL-8, which addressed the preceding 4 weeks). Additionally, in order to obviate recall error [8, 9], we assessed burden on the preceding day: in respect of HY, we enquired into duration and intensity of the headache, and lost productivity as a consequence of it.

### Diagnosis

Only one headache type was diagnosed in each participant (the most bothersome when more than one was identified).

According to the algorithm used to convert questionnaire responses to diagnoses [9], headache occurring on ≥15 days/month was first set aside from episodic headaches (frequency <15 days/month), and diagnosed according to reported acute medication consumption either as probable MOH (pMOH) or other headache on ≥15 days/month. To the episodic headaches, definite migraine criteria were first applied, then, in order, those of definite TTH, probable migraine and probable TTH [26]. For purposes of prevalence and burden estimations, definite and probable migraine were considered together as migraine, as were definite and probable TTH as TTH. A small minority of headaches remained unclassifiable.

When a participant reporting HY described one type of headache only, we assumed HY was of this type. Of those describing more than one type, we asked whether HY was of the same type as the most bothersome headache. Otherwise there was no diagnostic enquiry into HY, because this is problematic for a single episode.

### Analysis and statistics

Analyses were performed using SPSS/PC version 20.0 software packages for statistical analysis (SPSS, INC, Chicago, IL) and Excel Professional Plus 2010 Version 14.0.7166.5000.

We described categorical variables in terms of frequency (n) and proportions (%), with 95% confidence intervals (CIs) where appropriate. We summarised continuous variables as means ± standard deviations (SDs), with medians and quartiles when distributions were highly skewed. We recorded gender, age, employment, marital status and incomes of participants, the last in Lithuanian litas (LTL) which we converted to United States dollars (USD) at the exchange rate of the time (USD 1.00 = LTL 2.22). We recorded headache frequency in days/month, duration in hours (for HY only, this was categorized as <1, 1–4, 5–12 and >12 h), and intensity in terms meaning “mild”, “moderate” or “severe”, which we converted to a numerical rating scale of 1–3 (0 being no pain) and treated as continuous data. We calculated headache-attributed disability at individual level as the product of time in ictal state (itself a product of frequency and duration) and the disability weight (DW) from GBD2013 [29] for the disorder in question. We measured lost productivity from HALT in whole days/3 months, equating, according to accepted methodology [9, 27], “less than half achieved” to “nothing achieved” and counterbalancing this by equating “more than half” to “everything”. We calculated these losses as proportions of total productive time available. For HY, we reckoned productivity yesterday as either zero (less than half or nothing achieved) or 100% (more than half or everything achieved). We analysed WHOQoL-8 by summing the scores for the eight questions (each 1–5, higher scores indicating better QoL).

We calculated odds ratios (ORs) for gender- and age-related differences. We used chi-squared or Student’s *t* test (one-sided) to assess significance of differences. We regarded p < 0.05 as significant.

## Results

Of the 1137 pre-identified participants in the sample, all presumed to be eligible, 573 were available and willing and completed their interviews (participation proportion: 50.4%). There were more female participants (336 [58.6%]) than male (237 [41.4%]; chi-squared = 8.4448; *p* = 0.0037), necessitating gender-adjustments to observed prevalences. Mean age of participants was 40.9 ± 13.8 years; there was approximately even spread between 18 and 65, albeit with a non-significant preponderance (26.4%) among those aged 46–55 (OR: 1.3 [95% CI: 0.98–1.7]. Of those responding to the relevant questions, 62.8% were in paid employment or self-employment and 65.3% were married or living with a partner. The great majority (546 [95.3%]) were native Lithuanian speakers; only 27 (4.7%) were Russian-speaking, and only 12 of these (2.1%) were using Russian as their language at home.

Information on annual income was provided by 445 participants (77.7%). Of these, almost two thirds (292 [65.6%]) reported incomes below LTL 20,000 (USD 9000), including 170 (38.2%) below LTL 12,000 (USD 5400).

### Headache prevalence

Of the 573 participants, 497 reported headache ever in their lives (lifetime prevalence: 86.7% [83.7–89.3%]). Headache in the preceding year was reported by 437 participants (76.3%), more commonly by females than males (OR: 2.7 [1.8–3.9]; p < 0.0001) (Table 1). The gender-adjusted 1-year prevalence of any headache was 74.7%.

**Table 1:** Observed and gender-adjusted prevalences (1-year) of headache types by gender and age, and of headache yesterday (1-day)

	All headache* (*n* = 437)	Migraine (*n* = 117)	Tension-type headache (*n* = 240)	Probable MOH (*n* = 20)	Other headache on ≥15 d/m (*n* = 30)	Any headache yesterday (*n* = 70)
Observed prevalences (% [95% CI])						
All (*N* = 573)	76.3 [72.6–79.6]	20.4 [17.3–23.9]	41.9 [37.9–46.0]	3.5 [2.3–5.3]	5.2 [3.7–7.4]	12 2 [9.8–15.2]
Gender						
Male (*n* = 237)	65.8 [59.6–71.6]	9.7 [5.9–13.4]	43.9 [37.6–50.2]	1.7 [0.1–3.4]	5.9 [2.9–8.9]	7.2 [3.9–10.4]
Female (*n* = 336)	83.6 [79.3–83.2]	28.0 [23.2–32.8]	40.5 [35.2–45.8]	4.8 [2.5–7.1]	4.8 [2.5–7.1]	15.8 [11.9–19.7]
Age (years)						
18–25 (*n* = 108)	75.9 [67.1–83.0]	10.2 [4.5–15.9]	50.9 [41.5–60.3]	0.9 [0–2.7]	6.5 [1.9–11.2]	9.3 [3.8–14.8]
26–35 (*n* = 110)	83.6 [75.6–89.3]	24.5 [16.5–32.5]	50.9 [41.6–60.2]	0.9 [0–2.7]	1.8 [0–4.2]	9.1 [3.7–14.5]
36–45 (*n* = 108)	82.4 [74.1–88.4]	22.2 [14.3–30.0]	49.1 [39.7–58.5]	4.6 [0.7–8.6]	3.7 [0.1–7.2]	10.2 [4.5–15.9]
46–55 (*n* = 151)	76.2 [68.8–82.3]	26.5 [19.5–33.5]	32.5 [25.0–40.1]	6.0 [2.2–9.8]	7.3 [3.2–11.5]	14.6 [9.0–20.2]
56–65 (*n* = 95)	61.1 [51.0–70.2]	15.8 [8.5–23.1]	27.4 [18.4–36.4]	4.2 [0.2–8.2]	6.3 [1.4–11.2]	17.9 [10.2–25.6]
Gender-adjusted prevalences (% [95% CI])						
All (*N* = 573)	74.7 [71.0–78.1]	18.8 [15.9–22.3]	42.2 [38.3–46.3]	3.2 [2.0–4.9]	5.4 [3.8–7.6]	11.5 [9.2–14.4]

*Including unclassified headache (*n* = 30); *MOH* medication-overuse headache, *d/m* days/month

Prevalence of any headache was highest (83.6%) in those aged 26–45 years (Table 1). In comparison with all other ages, prevalence fell significantly (to 62.1%) after 55 years (OR: 0.43 [0.27–0.69]; *p* = 0.0014).

HY was reported by 70 participants (12.2%) (Table 1). Females were twice as likely as males to report HY (OR: 2.4 [1.4–4.3]; *p* = 0.0025). The gender-adjusted 1-day prevalence of any headache was 11.5%. Probability of HY increased steadily with age after 35 years, doubling by 56–65 years from just over 9 to 17.9% (Table 1; OR: 2.2 [1.1–4.3], *p* = 0.0307).

### Headache type

TTH (41.9%) was reported twice as commonly as migraine (20.4%); pMOH (3.5%) and other headache on ≥15 days/month (5.2%) were relatively uncommon (Table 1). There were 30 cases (5.2%) of unclassified headache among those characterised by headache on <15 days/month.

Table 1 also shows each headache type by gender and by age. Both migraine (OR: 3.6 [2.2–5.9]; p < 0.0001) and pMOH (OR: 2.9 [1.0–8.8]; *p* = 0.0588) were 3-fold more common among females than males, the latter not quite significantly. TTH (OR: 1.1 [0.8–1.6]) and other headache on ≥15 days/month (OR: 1.3 [0.6–2.6] were reported more commonly, but insignificantly so, by males. The gender-adjusted 1-year prevalences were 18.8% for migraine, 42.2% for TTH, 3.2% for pMOH and 5.4% for other headache on ≥15 days/month.

All headache types varied with age. Migraine increased in prevalence after age 25 years (OR: 2.9 [1.3–6.1]; *p* = 0.0066) and decreased again after 55 years (OR: 0.5 [0.3–1.0]; *p* = 0.0521). TTH declined with age from 35 years onwards, and was significantly less frequent after 55 years (OR: 0.4 [0.2–0.65]; *p* = 0.0007). pMOH showed a trend of increasing prevalence with age after 35 years (maximally among those aged 46–55 years: OR: 7.0 [0.9–55.8]; *p* = 0.0677). Numbers were too small to divide age groups by gender.

### Burden attributable to headache

#### Symptom burden

The mean frequency of all headache was 5.0 (median 2.5) days/month), mean intensity (on the scale 1–3) was 1.7 (mild to moderate), and mean duration was 10.0 (median 2.0) hours.

Table 2 shows “usual” frequency, intensity and duration by headache type and, for migraine and TTH, by gender. Migraine was reportedly more frequent than TTH (4.4 *versus* 2.7 days/month), and also more intense (mean 2.1 [moderate pain] *versus* 1.4 [mild pain]) and longer lasting (13.0 *versus* 6.7 h, although with wide variation). The only significant gender difference was in migraine duration (males 4.5 h, females 12.6; Student’s *t*-test, 2-sided: *p* = 0.0003).

**Table 2:** Symptom burden by headache type and, for migraine and tension-type headache, by gender

	All headache (*n* = 437)	Migraine (*n* = 117)			Tension-type headache (*n* = 240)			pMOH (*n* = 20)	Other headache on ≥15 d/m (*n* = 30)
		All	Male	Female	All	Male	Female		
Frequency (d/m)									
Mean ± SD	5.0 ± 6.5	4.4 ± 5.3	3.8 ± 2.8	4.6 ± 5.8	2.7 ± 3.7	2.7 ± 4.1	2.8 ± 3.4	18.5 ± 5.2	20.8 ± 6.0
Median	2.5	3.0	3.0	3.0	2.0	1.0	2.0	15.0	20.0
Quartiles 1, 3	1.0, 5.0	1.2, 5.3	2.0, 5.0	1.0, 5.6	0.8, 3.0	0.5, 3.0	1.0, 4.0	15.0, 20.0	15.0, 28.5
Intensity (scale 1–3)									
Mean ± SD	1.7 ± 0.7	2.1 ± 0.5	2.0 ± 0.4	2.1 ± 0.6	1.4 ± 0.5	1.4 ± 0.5	1.4 ± 0.5	2.4 ± 0.5	1.8 ± 0.5
Median	2	2	2	2	1	1	1	2	2
Quartiles 1, 3	1, 2	2, 2	2, 2	2, 2.5	1, 2	1, 2	1, 2	2, 3	1, 2
Duration (hr)									
Mean ± SD	10.0 ± 32.8	11.0 ± 16.3	4.5 ± 5.5	12.6 ± 17.7	6.7 ± 25.1	7.1 ± 34.4	6.5 ± 14.0	12.1 ± 9.8	9.1 ± 9.8
Median	2	4	2.75	5	2	2	2	11	3
Quartiles 1, 3	1, 6	2, 12	2, 3.75	2, 19.5	1, 3	1, 3	1, 4	3, 24	2, 24

*pMOH* probable medication-overuse headache; d/m: days/month

Both pMOH and other headache on ≥15 days/month were, of course, far more frequent (Table 2). Intensity of pMOH was moderate to severe (mean 2.4) and of other headache on ≥15 days/month somewhat less (mean 1.8), but both types were moderate in most cases (median 2).

#### Disability

Mean duration of all headache was 10.0 h. Coupled with a mean frequency of 5.0 days/month, this meant that, on average, people affected by headache spent 6.9% of their total time ([5.0/30]*[10.0/24]*100) in the ictal state (*ie*, with headache).

For migraine, these values were (4.4/30)*(11.0/24)*100, equating to 6.7%. Applying the disability weight (DW) of 0.441 for migraine from GBD2013 [29], we can calculate a mean disability per person with migraine of 3.0% (that is, each person loses the equivalent of 3.0/100 healthy life years). For TTH, with a DW of 0.037 [29], we can similarly calculate a much lower mean disability per person with the disorder of 0.09% ([2.7/30]*[6.7/24]*100*0.037). For pMOH (DW 0.217 [29]), estimated disability (based on small numbers) is much higher at 6.7% ([18.5/30]*[12.1/24]*100*0.217).

#### Loss productive time

The HALT questionnaire captured headache-attributed lost time from paid and household work (calculated as whole days: see Methods), and missed social occasions, during the preceding 3 months [27]. Table 3 shows the findings from this enquiry by headache type and gender.

**Table 3:** Lost productive time in whole days during the preceding 3 months, by headache type

	All headache (*n* = 437)		Migraine (*n* = 117)		Tension-type headache (*n* = 240)		Probable MOH (*n* = 20)		Other headache on ≥15 d/m (*n* = 30)	
	Male (*n* = 156)	Female (*n* = 281)	Male (*n* = 23)	Female (*n* = 94)	Male (*n* = 104)	Female (*n* = 136)	Male (*n* = 4)	Female (*n* = 16)	Male (*n* = 14)	Female (*n* = 16)
Lost paid workdays										
Mean ± SD	1.2 ± 4.5	1.5 ± 4.7	2.9 ± 7.9	2.3 ± 5.2	0.5 ± 2.9	0.5 ± 2.2	1.0 ± 2.0	6.1 ± 11.8	3.8 ± 6.6	2.2 ± 4.9
Median	0	0	0	0	0	0	0	0	0	0
Lost household workdays										
Mean ± SD	2.9 ± 8.8	3.7 ± 8.0	4.2 ± 7.6	4.5 ± 6.4	1.2 ± 3.0	1.6 ± 3.7	7.5 ± 15.0	15.6 ± 22.8	12.9 ± 23.2	7.4 ± 9.0
Median	0	0	0	2.3	0	0	0	6.0	6.0	5.0
Total mean lost workdays	4.1	5.2	7.1	6.8	1.7	2.1	8.5	21.7	16.7	9.6
Lost social occasions										
Mean ± SD	0.3 ± 1.7	0.4 ± 1.3	0.3 ± 0.8	0.7 ± 1.6	0.1 ± 0.3	0.1 ± 0.4	0	1.5 ± 2.8	1.9 ± 5.5	0.4 ± 1.0
Median	0	0	0	0	0	0	0	0	0	0

*MOH* medication-overuse headache; d/m: days/month

Here it should be noted that all but four of the 24 medians were 0, indicating on the one hand that most participants with headache lost no productive time and missed no social occasions and on the other that distributions among those who did were highly skewed, with especially badly-affected minorities. This said, mean total lost productive time from migraine was 7.3 days in males and 6.8 days in females; losses from TTH were much less (1.7 and 2.1 days respectively). Gender differences were generally small (numbers with pMOH and other headache on ≥15 days/month were too small to draw conclusions).

Assuming there were 65 (5*13) paid workdays in the preceding 3 months, the lost times from paid work attributable to migraine represented 4.5% (2.9/65) of all paid workdays in males and 3.5% (2.3/65) in females. Proportions lost from household work are less easily calculated: arguably there are 90 (3*30) potential days of household work in 3 months, but, if 65 of these are counted among paid workdays, it is also arguable that there are only 25. In the former case, losses were 4.7% (4.2/90) and 5.0% (4.5/90); in the latter 16.8% (4.2/25) and 18.0% (4.5/25). Lost times from paid work attributable to TTH were only 0.7% in both males and females. Much higher losses of 8–22 days were attributed to pMOH and other headache on ≥15 days/month. Lost times from paid work attributable to all headache were 1.8% (1.2/65) in males and 2.3% (1.5/65) in females.

Lost social occasions were few, although these numbers were dependent on social occasions being planned in the first place (data not collected).

### Headache yesterday

Of the 70 reports of HY, one third were in participants with pMOH (*n* = 10) or other headache on ≥15 days/month (*n* = 13). We could identify headache type in 37 others (migraine *n* = 19, TTH *n* = 18), either because the participants had only one headache type or because HY was typical of their most bothersome (and therefore diagnosed) headache. These numbers were insufficient to support burden estimates from HY by headache type.

HY was mostly moderate (mean intensity 1.9 on the scale 1–3) and mostly of short duration (<4 h), although 11 participants reported >12 h. Nevertheless, HY was associated with substantial lost productivity: 30 (42.9%) of the participants with HY achieved less than half, or nothing, of whatever they had planned. Counting yesterday in such cases as a wholly lost day, these amounted to an overall lost productivity yesterday of 5.2% (30/573). Of the 33 participants with HY for whom yesterday had been a workday, 10 (30.3%) had achieved less than half or nothing of what they had expected (four taking sick leave for the whole day). These cases amounted to lost productivity from paid work yesterday of 1.7% (10/573).

### Quality of life

Participants reporting no headache in the preceding year had a mean WHOQoL-8 summed score of 29.3 ± 5.8 (possible range 8–40). All headache types were associated with diminutions, which were significant for all but TTH. Participants with migraine scored 27.1 ± 6.0 (Student’s *t*-test, 2-sided: *p* = 0.0025 *versus* no headache), those with TTH 28.4 ± 6.0 (*p* = 0.126), those with pMOH 25.8 ± 4.8 (*p* = 0.0048) and those with other headache on ≥15 days/month 24.9 ± 4.8 (*p* < 0.0001).

## Discussion

As has been the case with all LTB studies, this population-based survey has revealed a lot of headache and much attributed burden. Almost three quarters (74.7%) of the Lithuanian population have a headache disorder of some type: migraine in 18.8% and TTH in 42.2%. These findings are considerably higher than global estimates of 14.7 and 20.8% from GBD studies [11–13], but within the ranges of studies using similar methodology [10] supported by LTB (15.6–25.6% for migraine, with China [9.3%] and Nepal [34.7%] outlying, and 20.6–44.6% for TTH, with China again outlying [10.8%]). We note that the GBD estimates are lowered by inclusion of studies using variable and sometimes questionable methods, and in some cases different diagnostic criteria [15]. In other words, these disorders are as common in Lithuania as everywhere else.

The relationships between headache and age are in line with expectations: both migraine and TTH are significantly less prevalent after 55 years. Migraine is strongly associated with female gender, again as expected. Migraine attacks reportedly lasted for much longer in females than in males (12.6 *versus* 4.5 h), a difference probably due largely to menstrually-related attacks. However, the medians were only 5 and 2.75 h respectively, and lower quartiles only 2 h in both genders, suggesting many attacks were truncated by treatment.

Notably, and of clear public-health concern, 3.2% of the population have pMOH. Although the prevalence of pMOH varies widely around the world, this is double the best global estimate of about 1.5% [30]; as will be seen, however, it is not an outlier. This disorder, too, is associated with female gender. More alarmingly, a total of 8.6% of the population have headache on ≥15 days/month – very high, but again not an outlier. These types of highly-frequent headache contribute substantially to the probability of HY and, in our sample, 11.5% reported HY. There was nothing special about “yesterday”: it was the day before the interviewer happened to call, and might have been any day since the interviewers worked all days of the week; therefore, this finding implies that, on any day in Lithuania, a similar proportion, about one person in nine, have headache.

To add to their public-health significance, all headache types except TTH are associated with significantly diminished QoL. They are the cause of a range of other ill-health burdens. Although headache was moderate overall, mean duration was 10.0 h. People affected by headache spend 6.9% of all their time – more than one day in every 15 – in the ictal state (*ie*, with headache). For migraine it is a similar 6.7%, with an attendant disability equivalent to the loss of 3.0/100 healthy life years. Disability from TTH is much lower (0.09%), but from pMOH it is, as expected (although this estimate is based on small numbers), much higher (equivalent to the loss of 6.7/100 healthy life years).

For migraine and pMOH, these are very important penalties in lost years, incurred by those affected. Furthermore, disability translates into lost productivity. While most participants lost no productive time due to headache, and missed no social occasions, there were badly-affected minorities whose losses were substantial. As a result, mean per-person lost times from paid work attributable to migraine were 4.5% in males and 3.5% in females. These are somewhat higher than the 3.0% disability estimate, and considerable. Proportions of time lost from household work were even higher (4.7–16.8% and 5.0–18.0% respectively, depending on how these were calculated), probably reflecting the more optional nature of household work compared with paid work. The finding that lost productivity exceeds disability estimated from time spent with headache is usual, and suggests that the disabling effect of migraine in fact outlasts headache.

These individual losses allow estimates of the corresponding losses at population level. Males with migraine, losing on average 4.5% of their paid worktime, and being 9.7% of all males aged 18–65 years (effectively the working population), consequently account for 0.44% (4.5%*9.7%) lost productivity among the entire male workforce. Females with migraine, being 28.0% of all females of this age, likewise account for 1.0% (3.5%*28.0%) lost productivity among the entire female workforce. If the two genders are in equal numbers among the workforce, the losses overall are 0.7%, which may translate directly into lost gross domestic product (GDP).

The other (household) work losses to migraine are similar if based on a denominator of 90 days: 0.46 and 1.4% among all males and all females respectively. But if the denominator is 25 days, they are 1.6 and 5.0%. It is never clear what exactly people include in “household work”, or how time engaged in it should be valued in comparison with paid worktime, but it certainly contributes to a nation’s economy.

Lost times at individual level due to TTH were much less, but to pMOH and other headache on ≥15 days/month much greater. At population level, lost paid worktime for those with TTH can likewise be estimated at 0.3% (0.7*42.2%) overall, and those for pMOH or other headache on ≥15 days/month (very approximately from small numbers) at 0.5%. In total, from all headache types, lost productivity among the entire workforce was 1.2% (1.8%*65.8%) for males and 1.9% (2.3%*83.6%) for females. The average of 1.5% is the total potential loss to GDP from all headache.

HY offers an alternative and corroborating means of estimation. In the first place, its reported prevalence of 12.2% can be directly compared with the predicted 1-day prevalence of headache, which is the product of the observed 1-year prevalence (76.3%) and the mean probability of headache on any particular day among those with a headache disorder. The latter is calculated from the mean frequency: 5.0 days/month indicates a probability of 0.17 (5.0/30). The product of 12.7% is highly compatible with the reported prevalence of HY, suggesting very little recall error in the reporting of frequency over the last 3 months. In the second place, we calculated lost productivity from paid work yesterday of 1.7%. As noted above, there was nothing special about “yesterday”, so this finding for HY seems likely to apply to every workday. This lost-productivity estimate is based on relatively small numbers, but its very close similarity to the 1.5% above generates confidence in the latter.

The limitations of this study are, to some extent, those of the Eurolight Project itself, which was “a very large and organizationally complex study, involving multiple collaborating partners (academic and lay) in ten countries [*making*] pragmatic methodological compromises in order to complete it” [31]. Eurolight also had considerable strengths: a questionnaire closely derived from the HARDSHIP questionnaire [9], and therefore validated in many different countries, cultures and translations; neutral screening questions expected to lead to better ascertainment than questions incorporating degrees of frequency or severity [8]; and diagnoses made according to a standard algorithm also widely used [9]. Nevertheless, Eurolight could not undertake further diagnostic validation in each of the translations it used, so diagnostic accuracy was not directly assessed. This was one limitation here, although we believe the instrument was reliable in view of its successful use elsewhere [9, 10], and cite, as evidence of this, the expected gender differential observed in migraine and pMOH but not TTH. The use of different sampling methods in Eurolight [22, 31] was not a limitation here, since sampling in Lithuania was population-based. While we sampled only from Kaunas region, Lithuania is a small and rather homogeneous country. More problematic was the rather small sample size of 1137, further diminished to 573 by participation of only 50.4%. Non-participants included those who had been selected but were not present on at least two visits to the household, and those declining to be interviewed (we did not seek reasons for refusal). The final value of N is above the 500 advised minimum for burden-of-headache studies [8], but the penalty is that estimates have relatively wide CIs. Additionally, although participation was much better in Lithuania than in Eurolight as a whole (27.5% on average, in those countries in which it was calculable [31]), there was still some probability of participation bias [8]. Nonetheless, the participating sample were representative for age, gender and rural/urban distribution of Lithuania’s population.

With these limitations in mind, which may not be so important to health policy, it is worth comparing Lithuania with Russia. This nearby country differs ethnically from the very much smaller Lithuania, but LTB’s nationwide population-based study in Russia used similar methodology and the HARDSHIP questionnaire [20, 21]. It was a larger study (*N* = 2,725), with a participating proportion of 74.3% (*n* = 2,025) [20]. The gender-adjusted 1-year prevalences were 20.8% for migraine (compared with 18.8% for Lithuania), 30.8% for TTH (42.2% for Lithuania) and 10.4% for all headache on ≥15 days/month (8.6% for Lithuania) [20]. Except for TTH, these differences are within the bounds of our uncertainty limits. The very high prevalence of headache on ≥15 days/month in Russia (10.4%) was largely behind the very high prevalence of HY (14.5%) [21]. A similar situation pertains in Lithuania: both these prevalences are only about 20% lower (8.6% and 12.2%). Where the countries differ is in pMOH: among the 10.4% in Russia with headache on ≥15 days/month, 68.1% were overusing acute medication [20], giving rise to an estimated prevalence of pMOH of 7.1% against Lithuania’s 3.2%. Cost of headache to GDP in Russia was estimated at 1.75% [32], similar to (given the uncertainty limits), and supporting, the 1.5–1.7% suggested in Lithuania.

We can also compare with Georgia [18, 19], although the study here (the first supported by LTB) uniquely used a different questionnaire [33]. Like Lithuania, Georgia is a small European country, formerly part of the USSR, but it otherwise differs from Lithuania historically, ethnically and culturally. Furthermore, unlike Lithuania, which has developed rapidly since 2000 to high-income status, Georgia has remained in the lower-middle income category of countries [34]. Georgia had a larger participating sample of 1145, of whom 15.6% were diagnosed with migraine, 37.3% with TTH and 7.6% with any headache on ≥15 days/month (including, perhaps, 0.9% with pMOH) [18]. These numbers are all lower than those in Lithuania, but (except for pMOH, which may have been underestimated in Georgia [unpublished data]) only by small and non-significant margins, and these may be attributable to the different diagnostic instrument. All three countries (Lithuania, Russia and Georgia) are remarkable for the high prevalence of headache on ≥15 days/month, which calls for investigation.

## Conclusions

Eurolight is not, generally, a source of reliable epidemiological data, and not designed to generate national estimates [31]. This population-based survey from Lithuania, however, was by far the most methodologically sound of the studies contributing to it. Despite the limitations mentioned, it does therefore provide a picture of headache in the country. As elsewhere, it is not a happy picture. The symptom, disability, lost-productivity and impaired-QoL burdens signal substantial ill-health, and therefore unmet need for health care [14]. This is a message that has been repeated to policy-makers in countries all over the world [34], but here, from this study, is empirical evidence to underpin it in Lithuania.

As in other countries, the high impact on individuals is matched by high economic burden; both are unmitigated by failures in health care. Health policy in Lithuania must take note of these findings, and of WHO’s advice that effective treatment of headache is desirable not only for its health benefits but also because it is likely to be cost-saving [35]. There are recommendations, for Europe, on the organisation of headache services to meet need [36].
